# Potential benefits of yeast *Saccharomyces* and their derivatives in dogs and cats: a review

**DOI:** 10.3389/fvets.2023.1279506

**Published:** 2023-10-25

**Authors:** Marta Maturana, Lorena Castillejos, Susana M. Martin-Orue, Anaelle Minel, Olivia Chetty, Ananda P. Felix, Achraf Adib Lesaux

**Affiliations:** ^1^Animal Nutrition and Welfare Service (SNiBA), Department of Animal and Food Science, Universitat Autònoma de Barcelona (UAB), Bellaterra, Spain; ^2^Department of Research & Development, Phileo by Lesaffre, Marcq-en-Baroeul, France; ^3^Department of Animal Science, Federal University of Paraná, Curitiba, Brazil

**Keywords:** companion animals, prebiotics, probiotics, postbiotics, yeast

## Abstract

Yeast *Saccharomyces* and its derivatives have been largely used in livestock and poultry nutrition for their potential positive impact on growth, performance, and general health. Originally included in animal diets as a source of protein, yeasts can also offer a wide range of by-products with interesting bioactive compounds that would confer uses beyond nutrition. Although its supplementation in livestock, poultry and even in humans is well documented, the available body of literature on the use of yeast and its derivatives in companion animals’ food, mainly dogs and cats’ diets, is still developing. Despite this, gut microbiota modulation, immune system enhancement or decreasing of potentially pathogenic microorganisms have been reported in pets when using these products, highlighting their possible role as probiotics, prebiotics, and postbiotics. This review attempts to provide the reader with a comprehensive on the effects of *Saccharomyces* and its derivatives in pets and the possible mechanisms that confer their functional properties.

## 1. Introduction

As the pet food industry keeps steadily growing annually, so does the demand for functional ingredients in dogs and cats’ diets. Owners look for ingredients that can provide additional health benefits beyond basic nutrition, echoing the functional foods trend already established in humans. Examples of these ingredients are omega-3 fatty acids, probiotics/prebiotics/postbiotics, or plants and vegetables’ extracts. Yeasts may also represent a good candidate. There are about 500 different yeasts species and some of them have been used both in humans and animals, especially livestock, for many years, *Saccharomyces cerevisiae* (*S. cerevisiae* or SC) being the predominant one. In this review, we will specifically focus on the use of *S. cerevisiae*, referring to it also as yeast.

Although the use of yeast in livestock and poultry feed has been widely reviewed, there is still a lack of information about the effects on dogs and cats, admittedly the number of studies is increasing. Moreover, despite the many beneficial health effects reported, the supplementation of yeast-based products has not always resulted in consistent effects, and the precise mode of action of these compounds has been difficult to determine. Thus, the aim of this review is to provide an overview on the use of *Saccharomyces* and/or their derivatives, as a functional ingredient in pet food, discussing its general possible modes of action with a specific focus on *in vivo* studies in companion animals.

## 2. *Saccharomyces* composition and products

Yeasts cells intracellular composition includes amino acids, peptides, carbohydrates, salts, vitamins, monosodium glutamate, nucleic acids, enzymes, and cofactors. On the other hand, yeast cell wall (YCW) is mostly formed by Beta-glucans (β-glucans), glycoproteins, and mannan oligosaccharides (MOS), and to a lesser extent by chitin. There are different forms of yeasts being studied, ranging from viable yeast cells to purified components of the cell wall, as shown in Table 1. From primary yeast cultures that undergo propagation and fermentation steps, yeasts by-products are obtained via downstream processing (1, 2). Live yeast cells can be inoculated in specific culture media to ferment it and subsequently be dried, forming a yeast culture containing a wide variety of metabolic products. Yeast cells can also undergo a process of hydrolysis or autolysis to obtain yeast lysates that contain both intracellular and cell wall fractions. However, intracellular components and carbohydrates of the cell wall can also be found separately as yeast extract and cell wall products, respectively.

**Table 1:** Most common *Saccharomyces cerevisiae* products added in animal feed, with its production process and the major composition (1).

Product	Process	Composition
Dried yeast (also dehydrated primary yeast)	Drying of whole yeast cells (high temperature).	Biomass of dead yeast cells.
Active dry yeast (also dehydrated active yeast or live yeast)	Drying of whole yeast cells.	Live yeast cells.
Yeast culture (also dehydrated yeast culture or SCFP in the case of *Saccharomyces cerevisiae*)	Inoculation of live yeast cells in a specific culture medium followed by controlled fermentation and drying.	Yeast biomass (residual viable cells, dead cells, and yeast cell wall fragments), fermentation metabolites, and residual growth medium.
Lysate yeast (also hydrolyzed yeast /hydrolysates or autolyzed yeast/autolysates)	Hydrolysis or autolysis of whole yeast cells.	B vitamins, peptides, amino acids, glutamic acid, nucleotides, minerals, mannan oligosaccharides, and β-glucans.
Yeast cell wall components	Centrifugation after the hydrolysis/autolysis of the yeast.	Mannan oligosaccharides and β-glucans (together or individualized).
Yeast extract	Centrifugation after the hydrolysis/autolysis of the yeast.	B vitamins, peptides, amino acids, glutamic acid, nucleotides, and minerals.

SCFP, *Saccharomyces cerevisiae* fermentation product.

Most of the yeast-based products available in the European market are registered under the European Food Safety Authority (EFSA) category of zootechnical additives, and subcategories of gut microbiota stabilizers or digestibility enhancers, depending on the product and target species. Whereas dried yeast is used for its nutritional value as a specialty amino acid, vitamin and mineral supplement, the rest of the products could fall under the definitions of probiotics, prebiotics, and postbiotics established by the International Scientific Association for Probiotics and Prebiotics (ISAPP). Active dry yeast is formed by live yeast cells and acts as a probiotic, defined by ISAPP as “live microorganisms that when administered in adequate amounts, confer a health benefit on the host” (3). The probiotic role of *Saccharomyces cerevisiae* var. *boulardii* (*S. boulardii*), a strain of the same genus as SC, nearly identical at a molecular level but physiologically different (4), is well established. However the probiotic role of *S. cerevisiae* needs further investigation, although some recent studies in dogs reported positive and promising results on modulation of fermentative metabolites and microbiota composition and function (5). On the other hand, yeast lysates, yeast extracts, and YCW components, mostly MOS and β-glucans, could be considered prebiotics and their use is more extended, with more studies reporting benefits on both human and animal health (1, 6). Prebiotics are defined by the ISAPP as “a substrate that is selectively utilized by host microorganisms conferring a health benefit” (7). Lastly, yeast culture, also known as *Saccharomyces cerevisiae* fermentation product (SCFP), would pertain to the still recent term “postbiotics” defined by the ISAPP as “preparation of inanimate microorganisms and/or their components that confers a health benefit on the host” (8).

## 3. *Saccharomyces* as a functional ingredient: modes of action

### 3.1. Microbiota improvement

A balanced gut microbiome is of vital importance for the health of the host and yeasts have been proven to cause shifts in the bacteria population of the gastrointestinal tract in several species (9, 10). *In vitro* studies reported that the association of some strains of *Saccharomyces* and *Lactobacillus rhamnosus* exert a protective effect on human microbiota following antibiotic administration (11) or against pathogenic bacteria (12). The use of yeast β-glucan also promoted the growth of *Bifidobacterium longum* and other health-promoting gut microbiota in a study simulating human saliva, gastric and intestinal digestion (13). Similarly, mannan and MOS derived from yeast were able to grow specific lactic acid bacteria strains *in vitro* and beneficial *Bacteroides* in humans (14, 15). Thus, yeast and yeast-based products may potentially promote host’s gut health.

### 3.2. Immune system enhancement

The supplementation of yeast products can modulate the immune system through changes in the innate and adaptative response. It is thought that the main responsible of these properties are the β-glucan and MOS present in yeast. The specific changes are different depending mainly on the type of product and the general health status of the animals. As reviewed by many authors, yeast products can affect concentrations of platelets and WBC populations, phagocytic capacity of cells, secretion of pro- and anti-inflammatory cytokines, acute phase proteins concentrations, and antibodies production by binding with complement receptor type three (CR3), dectin-1 or TLRs on different cells (16–18). Yeasts may also have an indirect effect on the immune system through microbiota; as previously mentioned, β-glucans and MOS could increase *Bifidobacterium* population which, in return, has been shown to decrease the expression of toll-like receptors (TLRs), NFkB, and cytokines, *in vitro* and *in vivo* with mice (19–21). Moreover, emerging studies also point out the potential of yeast of altering the metabolism, including availability of glucose and fatty acids, as another factor contributing to their capacity of modulate the immune system (16). In conclusion, the immune response is a complex process that goes beyond the response of a single immune parameter; thus, to truly understand the mechanisms behind the possible beneficial effects of the use of yeast as an immunomodulator, integration of the different factors is needed.

### 3.3. Increased digestibility

Glucans present in yeasts could improve nutrient digestibility through the modification of passage rate in the small intestine. This effect would be mediated by the formation of a semisolid gel promoted by yeast’s alkali-soluble glucans that could slow the passage and increase the time that the chyme is in contact with enzymes, resulting in a higher digestibility of nutrients (22). As a result of this, bacterial fermentation in the large intestine would be reduced as less fermentable substrate would be available. However, one should be cautious with this hypothesis as *in vivo* studies with yeast β-glucans have reported increases in apparent total tract digestibility (ATTD) in pre-ruminant calves (23) or no changes at all in dogs (24). Other possible mechanism of yeast to modulate nutrient digestibility is the production of enzymes, such as amylases, lipases, and proteases. Some studies with fish and hens have reported increases in digestive enzymes with yeast supplementation, resulting in improved nutrients digestibility (25, 26).

Despite this, other studies have reported decreases in crude protein (CP), nitrogen-free extracts and dry matter (DM) apparent digestibility in dogs supplemented with MOS (27). These reductions in digestibility were accompanied by increased total fecal water and decreased unbound water which could mean different physical properties of intestinal chyme. Thus, the higher water binding could have had an impact on the solubility of nutrients and affected digestibility.

### 3.4. Protection against toxins and pathogens

*Saccharomyces cerevisiae* and other genetically close yeasts can exert a protective effect against toxins and pathogens through different mechanisms that involve the binding of bacteria and toxins, enhancement of immunoglobulins production by the host, and releasing of proteases that destroy certain toxins, among others (28, 29).

One of the most frequent protection mechanisms of yeasts that can be found in the literature is the capacity of binding pathogenic bacteria, avoiding its union with the host’s receptors, being MOS the main responsibles for this binding activity. Tiago et al. (30) showed both *in vivo* with mice and *in vitro* that selected strains of *S. cerevisiae* were able to capture some enteropathogenic bacteria on the surface, regardless of the yeast viability. However, this capturing ability was limited to specific Gram-negative enteropathogens such as *Salmonella* and *Escherichia coli* (*E. coli*). Mannan oligosaccharides form part of *Saccharomyces* cell walls, and their concentration is especially high in yeast derivatives such as YCW. The mechanism would be that mannose can occupy binding sites on fimbrial adhesins on the surface of bacteria. This way, mannose residues in live yeast or derivatives could prevent certain bacteria that possess fimbria type-1 from adhering to the intestinal mucosa, thus avoiding colonization and multiplication to disease-causing levels (31–33).

*Saccharomyces cerevisiae* var. *boulardii* has been widely used in treating *Clostridium difficile* diarrhea and colitis. The mechanisms of this effect include increasing the activity of intestinal disaccharidases (34) and stimulation of the intestinal secretion of s-IgA and of the secretory component of immunoglobulins (35, 36). Other authors characterized a serine protease released by the yeast that hydrolyzes toxins A and B, which are *C. difficile*’s mediators of diarrhea and colitis, thus inhibiting the binding of the toxins to its intestinal receptor (37–39). More recent studies have found that *S. boulardii* exerts an effect on the structural component of the microbe’s biofilm matrix *in vitro*, possibly affecting intestinal colonization (40). Reports about the use of *S. cerevisiae* against this pathogen are scarce but some demonstrate a protective effect of certain strains of the yeast in rats and mice (41, 42). Inhibition of other toxins such as cholera toxin or aflatoxin B1 has also been observed in *in vitro* and *in vivo* trials using rats, with *S. cerevisiae* and *S. boulardii*, hypothesizing that in this case the mode of action included binding of the toxin through a receptor located in its surface (43, 44).

*Saccharomyces cerevisiae* was also able to reduce heat-labile enterotoxins production from *Enterotoxigenic Escherichia coli* (ETEC), associated with the removal of the toxin by the yeast and the reduction in the expression of its corresponding gene *eltB* (45). In the same series of assays, the yeast also interacted directly with ETEC through agglutination in a mannose-related manner.

Lastly, *S. cerevisiae*-based probiotic has shown benefits in the treatment of bacterial vaginosis produced by *Gardnerella vaginalis* in mice (46). In this case, the suggested mechanisms of action include direct interference with adherence to vaginal tissues, inhibition of sialidase activity and reduction of vaginal epithelial exfoliation.

### 3.5. Antioxidant effect

The activity of *S. cerevisiae* and derivatives has been assessed to explore their potential as natural antioxidants, protecting cells against oxidative stress. Although it is probably a result of the combined action of different factors, cell wall β-glucans and mannans are thought to be the main actors in the antioxidant capacity of yeasts. As widely review in the literature, these compounds can boost the antioxidant status of the body by improving the activity of antioxidant enzymes such as glutathione peroxidase (GPx), catalase or superoxide dismutase (SOD) (17). Experiments with porcine jejunum epithelial cell lines damaged by deoxynivalenol, a strong inducer of oxidative stress, reported promising results. The association of mannan and β-glucan from yeast cell walls was able to down-regulate the production of malondialdehyde (MDA) and reactive oxygen species (ROS), while up-regulating the production of glutathione (47). *In vivo* studies with weaned piglets, supplemented with *S. cerevisiae* cell wall, also resulted in decreased serum MDA and increased activity of the enzymes GPx, catalase and SOD (48). The addition of yeast β-glucans in broilers exposed to mycotoxins improved serum levels of antioxidant enzymes and lower lipid peroxidation as well (49). Moreover, the naturally good antioxidant capacities of selenium seem to be improved by the enrichment with yeast, due to a synergic effect, as shown in *in vitro* and *in vivo* assays with mice (50).

### 3.6. Other effects

#### 3.6.1. Skin health

*Saccharomyces cerevisiae* extract, known occasionally as “skin respiratory factor,” has largely been used by humans for the treatment of burns, wounds and hemorrhoids (51). Studies with human and animal models indicate that it accelerates healing stages: inflammation, angiogenesis, granulation tissue and epithelial migration (52–55). The active fraction of the extract has been identified as a mixture of peptides from 6 to 17 kD formed by: copper zinc superoxide-dismutase ubiquitin, glucose-lipid regulated protein and acyl-CoA binding protein (51, 52). Gruenstein et al. (56) used a human monocyte-derived cell line, THP-1, to study the mechanisms involved in the extract and wound healing. These cells are widely used to study monocyte functions and mechanisms, being monocytes one of the first immune cells to reach the wound site. Apparently, the yeast derivative activates phospholipase-C which results in an increase of cytoplasmic Ca^2+^. This in turn initiates the transcription of early genes for tissue repair, such as c-fos. β-glucans are other components of the yeast cell wall that can have an effect on wound healing, facilitating the transportation of macrophages to the wound site, stimulating the tissue granulation and re-epithelization, and improving collagen deposition (57).

Topical application of *S. cerevisiae* has been shown to improve the healing of burn wounds and to enhance the action of a collagen hydrogel-scaffold, increasing epithelialization and biomechanical performance of wound area and reducing the scar size, in rats (58). Studies with seabreams and trouts showed that feed supplementation with *S. cerevisiae* improved skin health and skin recovery from wounds (59, 60).

The evidence of *S. cerevisiae* wound healing effect and its antioxidant properties have targeted the yeast as a potential biotechnological raw material in dermo-cosmetic formulations. Dermatological formulations that include *S. cerevisiae* extract or derivatives showed positive effects in skin moisture and roughness in animal and human models (15, 57, 61); protection against UV irradiation both *in vitro* and in humans (62, 63) and itch relief in chronic pruritus in humans (64, 65).

*Saccharomyces cerevisiae* has also been used as a skin cell model in experimental biology. Lastly, this yeast has been linked to different skin diseases such as psoriasis or atopic dermatitis playing a pro- or anti-inflammatory effect, but the exact mechanism has yet to be determined (66).

#### 3.6.2. Mood modulator

Probiotic ingestion has been linked to reduced anxiety-like behaviors via the hypothalamic–pituitary–adrenal axis and the gut-brain axis (67). Supplementing a mixture of *Bifidobacterium lactis*, *Lactobacillus acidophilus*, and *Lactobacillus fermentum* to rats exposed to noise stress decreased behaviors related to anxiety and also serum corticosterone (68). *Saccharomyces* sp. has also been proven to attenuate anxiety-like behaviors in rats exposed to LPS, possibly through modulation of the gut microbiota and hypothalamic–pituitary–adrenal axis (69). In this previous study, yeast supplementation led to significantly lower serum levels of cortisol and corticosterone. Supplementation prevented the reduction in the serum levels of serotonin and brain-derived neurotrophic factor (downregulated during stress) in rats challenged with LPS, compared with those not supplemented and challenged; however, the difference was not significant. Healthy medical students presented a decreased salivary serotonin concentration when facing a stressful event after 30-day supplementation with *Saccharomyces* sp., although an association with anxiety, depression or eating attitudes was not found (70). Hemiplegic spastic cerebral palsy rats that exhibited depression behavior improved their mental state when a *Saccharomyces* sp. treatment was applied. This yeast increased gut microbiota diversity and decreased the level of inflammation and the hypothalamic–pituitary–adrenal axis activity (71). In a study with healthy human volunteers, subjects consuming the *S. cerevisiae* yeast hydrolysate showed better marks in questionnaires that assessed stress and anxiety, suggesting that this derivative could be effective in modulating behavior (72).

## 4. *Saccharomyces* in dogs and cats

There are limited publications examining the impact of yeast or yeast by-products in the dog and cat, although the number is increasing. This contrasts with livestock, where there are a wide number of publications on the use of yeast or yeast by-products. Nevertheless, there is an ever-increasing demand for functional ingredients that improve companion animal health and yeast may prove to provide such benefits.

Pets could benefit from the inclusion of yeast products in their diet, as some promising results have been described in the scientific literature including the modulation of the intestinal microbiota (with potential increases in *Bifidobacterium* or *Lactobacillus*), enhancing of the immune function, reduction of potentially pathogenic microorganisms such as *E. coli*, improvement of blood parameters related with obesity and possible improvement in the antioxidant status, among others. In the following sections we review some of the most recent evidence on the potential benefits of using yeast or derivatives as functional ingredients in petfood. The main outputs of studies with live yeast *S. cerevisiae* and its by-products in dogs and cats are shown in Tables 2, 3, respectively.

**Table 2:** Main outputs of different studies on the use of live yeast *Saccharomyces cerevisiae* and by-products in dogs.

Product	Dose	Effects of the yeast product	Ref.
LY SC (strain CNCM I-5660; Actisaf®; Phileo by Lesaffre)	0 (CON) or 0.12 (SUP) g daily of LY SC (1 × 10^10^ CFU/g) for 49 days with abrupt change of diet at day 22. *Abrupt dietary change as challenge	Fecal microbiota: - ↓ Dysbiosis index, ↑ abundance of *Bifidobacterium* (days 35 and 49) and *Turicibacter*, and ↓ abundance of *Lactobacillus* and *E. coli.* - ↓ Firmicutes and ↑ Actinobacteria after the challenge. Hindgut fermentation products (fecal): - ↓ Ammonia (day 23), ↓ total biogenic amines (days 21 and 49), and aromatic compounds. ↑ butyrate concentration. - Others: - CON showed upregulation in genes related to virulence factors, antibiotic resistance, and osmotic stress.	(5)
LY SC (strain MUCL 39885; Biosprint®, Prosol S.p.A.)	0 (CON) or 7 × 10^10^ CFU SC/kg feed daily for 35 days (SUP)	Digestion: - ↓ Fecal DM; the magnitude of the effect is questionable. - =BW and feed intake.	(73)
LY SC (strain CNCM I-4407; Actisaf Sc 47; Phileo by Lesaffre)	0 (CON) or 2.9 × 10^8^ CFU SC/kg BW (SUP) daily for 42 days	Fecal microbiota: - ↓ *E. coli* and enterococci counts. - Digestion: - ↑ Weight gain- - ↑ ATTD of NDF, but not of the rest of nutrients.	(74)
Different fermentation media with SC	0 (CON), 10 g/kg food brewer’s yeast (SUP1), 10 g/kg food brewer’s yeast + corn yeast (SUP2) and 10 g/kg food brewer’s yeast + corn yeast + cell wall fractions (SUP3) for 20-day periods.	Fecal microbiota: - ↑ *Bacteroides, Faecalibacterium, Phascolarctobacterium, Coprococcus.* Hindgut fermentation products (fecal): - ↑ Total SCFA, acetate and butyrate. - ↓ Indole. Digestion: - ↑ Preference.	(75)
SCFP (TruMune, Diamond V Mills, Inc.)	0 (CON) or 250 mg SCFP daily (equivalent to approximately. 8.6 mg/kg BW) (SUP) for 10 weeks *Exercise challenge*	Fecal microbiota: - After 21 days of supplementation, before the first exercise challenge, ↑ *Clostridium* in CON whereas not change in SUP. Others: - For the rest of the parameters, exercise challenge was the primary cause of the shifts observed, but not the yeast.	(76)
SCFP (TruMune, Diamond V Mills, Inc.)	0 (CON) or 30 mg SCFP/kg BW (SUP) daily for 11-weeks periods. *Dogs challenged with transport stress	Digestion: - Change in fecal DM: ↓ (tendency) in CON but stable in SUP after transport. Antioxidant properties: - Change in serum malondyaldehide ↑ and in serum 8-isoprostane ↑ (tendency), after transport, whereas in CON ↓. - No changes in the expression. Of genes associated with blood cyclooxygenase-2 and myeloperoxidase mRNA, after transport, whereas in CON ↑.	(77)
SCFP (Diamond V Mills, Inc.)	0 (CON) or 30 mg SCFP/kg BW (SUP) daily for 10-weeks periods.	Inmmunomodulation: - ↓ Changes in unstimulated lymphocytes and stimulated IFN-γ secreting T cells. - ↓ Platelets; variable in CON. - Variable basophils; ↑ in CON. - Antioxidant properties: - Change in serum superoxide dismutase concentration was ↑ and in catalase mRNA expression was ↓. Skin: - ↑ (tendency) sebum concentration. - ↑ Transepidermal water loss values in back region of CON and in ear region of SUP.	(78)
SCFP (XPC™, Diamond V Mills, Inc.)	0% (CON), 0.3% (SUP1), or 0.6% (SUP2) SCFP of diet for 25-day periods.	Fecal microbiota: - ↑ Actinobacteria and Firmicutes, and ↓ Fusobacteria. - ↓ *Allobaculum* and *Fusobacterium*, and ↑ *Clostridium*. - ↑ *Collinsella* and ↓ *Prevotella* in SUP2. Hindgut fermentation products (fecal): - ↑ Propionate. Immunomodulation: - ↑ phagocytosis index- Digestion: - CF ATTD, ↓ CP and NFE ATTD, and ↓ ME in supplemented groups.	(79)
SCFP (Diamond V Mills, Inc.)	0 (CON), 250 mg (SUP1) or 500 mg (SUP2) daily for 14 weeks. * Dogs underwent exercise and transport stress	Immunomodulation: - ↑ TNF-α, ↓ IgE and IgG. - ↑ Haptogoblin, serum amyloid A, and C-reactive protein Antioxidant properties: - ↓ Thiobarbituric acid reactive substances, creatine kinase, and ↑ total antioxidant capacity. Others: - ↑ Activity, running speed, and stool quality.	(80)
SCFP (Diamond V Mills, Inc.)	0 (CON), 125 (SUP1), 250 (SUP2), or 500 (SUP3) mg SCFP daily for 28-day periods (12.1, 24.3, and 48.5 mg/kg BW) Sub-study: palatability test, 0.2% SCFP of diet.	Fecal microbiota: - ↑ Actinobacteria, ↑ (tendency) Firmicutes, ↓ Fusobacteria. - ↑ *Bifidobacterium*, ↑ (tendency) *Prevotella*, and ↓ *Fusobacterium*. Hindgut fermentation products (fecal): - ↓ Phenol and phenol+indoles, linearly with dosage. - Immunomodulation: - ↓ WBC with increasing SCFP dose; SUP2 had ↓ WBC than CON. - ↑ MHC-II + B cell and monocyte expressing surface MHC-II populations. - ↓ TNF-α secretion from cells stimulated with TLR2, TLR3, TLR4 and TLR7/8 agonists. - ↑ IgE with dosage. - CON had the highest TNF-α concentrations. Digestion: - Slightly softer when comparing all SCFP treatments with CON. - TTD was not influenced. - ↑ Preference.	(81)
Hydrolyzed SC (Progut®, Suomen Rehu, patented EP 1387620 hydrolysis process)	0% (CON) and 0.3% (SUP) hydrolyzed SC of diet for 14 days (day 0 to day 14). Whole study 42 days.	Fecal microbiota: - ↑ *Bifidobacterium* (day 14 and day 42), lactic acid bacteria (day 42) and *Clostridium* (day 42). Hindgut fermentation products (fecal): - ↑ pH (day 28). Obesity: - ↓ Serum lipid profile (triglyceride, cholesterol) (day 42). Kidney and liver: - ↑ ALT (day 14), AST (day 28 and 42), and urea serum levels.	(82)
Hydrolyzed SC (Brewer’s Yeast, BY)	Control diet (CON) vs. control diet substituted by 15% BY (SUP-BY), 7.5 and 15% autolyzed sugar cane (ASCY) or integral sugar cane (ISCY). 10 days.	Digestion: - ↓ Gross energy ATTD in ASCY and ISCY compared to CON. - ↓ Fecal DM with the addition of yeast. - ↑ Preference.	(83)
Yeast extract (Nupro®, Alltech Brasil Ltda.)	0 (CON), 40, 80, or 120 g yeast extract/kg diet daily for 10-day periods Sub-study: palatability test, 0 vs. 40 g yeast extract/kg diet.	Hindgut fermentation products (fecal): - ↑ Total SCFA, propionate and butyrate with dosage. - ↓ Isovalerate and pH with dosage. Digestion: - ↓ ATTD of CP and AHEE with dosage. - ↑ Preference.	(84)
YCW (Biolex MB 40, Leiber GmbH, Bramsche, Germany)	*In vitro* study, simulating the canine gastrointestinal tract. Doses: 0 (CON), 0.5 (SUP1), 1.0 (SUP2) and 2.0 (SUP3) g/d corresponding to 0.05, 0.1 and 0.2% in pet food, for 3 weeks.	Fecal microbiota: - ↑ Bacteroidetes and ↓ Fusobacteria. - ↓ Enterobacteriaceae and Fusobacteriaceae. Hindgut fermentation products (fecal): - ↑ Gas production. - ↑ Acetate and butyrate. - ↑ Propionate for all SUP groups compared to CON in PC, but only for SUP3 in DC. - BCFA were low overall but significantly higher for SUP1 and SUP2 in both PC and DC, and only for SUP2 in DC. - ↑ Ammonia for SUP2 in PC and DC.	(85)
YCW (Safmannan®, Phileo by Lesaffre)	0 (CON) or 365 mg yeast daily (0.2% of diet) (SUP) for 28-day periods with an abrupt diet transition at day 15 *Abrupt dietary change as a challenge.	Fecal microbiota: - ↑ (tendency) *C. perfringens*. Immunomodulation: - ↑ (tendency) fecal IgA, after abrupt diet transition.	(86)
Conventional YCW and specific high solubility of MOS YCW (YCWs) (Biorigin, Brazil)	0 (CON), 0.3% YCW (SUP1) and 0.3% YCWs (SUP2) of the diet for 32-day periods	Hindgut fermentation products (fecal): - ↑ Butyrate and ↓ lactate in SUP2. - ↑ Putrescine and ↑ (tendency) spermidine in SUP2. Immunomodulation: - ↓ Serum IL-6 in SUP2. - ↓ (tendency) serum IL-6 and TNF-α in SUP1. - ↑ Phagocytic index in peripheral monocytes. Digestion: - ↓ AHEE ATTD in SUP2.	(87)
YCW (DMB; Bio-Mos, Alltech Nicholasville, KY)	0 (CON) and 1.4% YCW (SUP) of the diet for 21-day periods *Raw diets: beef and chicken.	Fecal microbiota: - ↑ *Bifidobacterium* and ↓ *Clostridium celerecrescens.*	(88)
YCW (DMB; Bio-Mos, Alltech Nicholasville, KY)	0 (CON) and 1.4% YCW (SUP) of the diet for 21-day periods *Raw diets: beef and chicken.	Hindgut fermentation products (fecal): - When used in the chicken diet: ↓ pH, ↓ indole and ↑ spermine. - When used in the beef diet: ↑ fecal acetate, total SCFA, and spermine. Digestion: - When used in the beef diet: ↑ (tendency) fecal output and softer feces.↓ DM, OM, CP and energy ATTD.	(89)
YCW (Safmannan®, Phileo by Lesaffre)	0 (CON), 0.07 (SUP1), 0.35 (SUP2) or 0.91 (SUP3) g of YCW per animal twice daily for 14-day periods. Doses correspond to 0, 0.05, 0.25, 0.45, or 0.65% of food offered.	Fecal microbiota: - ↓ *E. coli*. *Clostridium perfringens* responded cubically to supplementation. Immunomodulation: - ↓ (tendency) quadratically of WBC and eosinophils, and ↓ linearly of monocyte with dosage. - Serum IgA responded cubically to supplementation whereas ileal IgA tended to respond quadratically, with the greatest value in SUP2. Digestion: - ↑ (tendency) apparent Ileal digestibility of DM, OM, CP, and GE. - ATTD of DM, OM, CP, AHEE, and GE responded cubically to supplementation, being lowest in SUP2.	(90)
Yeast-derived MOS, high in mannoproteins (Actigen, Alltech Inc.)	0 (CON), 400 mg (SUP1), or 800 mg MOS/kg (SUP2) of diet daily for 28 days, *Use of adult and elderly dogs	Immunomodulation: - ↑ (tendency) neutrophil phagocytic activity in SUP1 compared with SUP2. Antioxidant properties: - ↑ (tendency) production of H_2_O_2_ when neutrophils were stimulated with LPS. Skin: - Diet influenced the delayed cutaneous hypersensitivity test upon saline inoculation.	(91)
Yeast-derived MOS (Provimi Animal Nutrition)	0 (CON) or 15 g MOS/kg of diet (SUP) for 150 days.	Immunomodulation: - ↑ Cell-mediated immune response, CD4+ lymphocyte population and CD4+:CD8+ ratio. Obesity: - ↓ Serum total- and LDL- cholesterol.	(92)
Yeast-derived MOS (Bio-MOS, Alltech Inc.)	0 (CON) or 2 g/kg BW (SUP) daily for 20 days from 24 h after infection. *Challenge with enteropathogenic Escherichia coli	Management of gastrointestinal disorders: - Faster recovery from infection.	(93)
Yeast-derived MOS	0 (CON), 1% dietary chicory (SUP1), 1% dietary MOS (SUP2) or 1% chicory +1% MOS (SUP3) for 28 days.	Fecal microbiota: - ↑ *Bifidobacterium* in SUP1 and SUP2. - ↓ *E.coli* in SUP2 Immunomodulation: - ↓ (tendency) lymphocytes in SUP2 and SUP3. - ↑ (tendency) neutrophils in SUP1 and SUP3. Digestion: - ↑ (tendency) feed intake in SUP2 and SUP3. - ↑ Fecal score in SUP3, although still in desirable range.	(94)
Yeast-derived MOS (Bio-MOS, Alltech Inc.)	0 FOS/MOS (CON), 1 g FOS (SUP1), 1 g MOS (SUP2), 1 g FOS + 1 g MOS (SUP3) twice daily for 14-day periods	Fecal microbiota: - ↓ Total aerobes in SUP2. - ↓ (tendency) total anaerobes in SUP3. Hindgut fermentation products (fecal): - ↓ Phenols + indoles, and ↓ (tendency) indoles in SUP1 and SUP3. - ↑ (tendency) pH in SUP2. Immunomodulation: - ↑ Ileal IgA in SUP3. - ↑ lymphocytes in SUP2.	(95)
Yeast-derived MOS (Bio-MOS, Alltech Inc.)	0 (CON) vs. 1 g/kg BW (SUP) daily for 10 days (5% of diet)	Hindgut fermentation products (fecal): - ↓ Unbound water, fecal pH, and ammonia. Digestion: - ↓ ATTD ofCP, DM and nitrogen-free extracts. ↑ ATTD of CF.	(27)
Yeast-derived MOS (DP607, Alltech Inc.)	0 (CON) vs. 5 g/kg of diet DM (SUP) daily for 21-day periods.	Hindgut fermentation products (fecal): - ↑ (tendency) ileal butyrate in CON. Digestion: - ↓ (tendency) amount of feces excreted on a DM in CON.	(96)
Concentrated BY (not specified, possibly β-glucans) (F.L. Emmert Company, USA).	0 (CON) vs. 7 mg/kg BW of β-glucan (SUP) daily for 10 weeks.	Hindgut fermentation products (fecal): - ↑ Arabinose. Immunomodulation: - ↓ (tendency) gut permeability. - ↓ Serum haptoglobin levels.	(97)
Yeast-derived β-glucan	0 (CON) vs. 0.1% (SUP) of diet, for 90 days, in obese dogs.	Obesity: - ↓ Plasma glucose triglyceride, and cholesterol concentrations. - ↑ GLP-1. Immunomodulation: - ↓ TNF-α.	(98)
Yeast-derived β-glucan (Wellmune for Pet™, Kerry Incorporated, Beloit, WI)	0 (CON), 0.012 (SUP1) and 0.023% (SUP2) of diet for 28 days.	- No effect of the β-glucan in the analyzed parameters.	(24)
Yeast-derived β-glucan	0 (CON), 150 ppm β-glucan top-dressed (SUP1), 150 ppm retorted β-glucan included in diet formulation (SUP2), during 42 d. *Vaccine challenge*	Digestion: - ↑ ATTD of DM, CP and GEin SUP2. ATTD of GE was greater for SUP2 than for both CON and SUP1.	(99)
Yeast-derived β-glucan (BG01 and BG02, Biorigin)	0 (CON), 15 mg/kg/day of BG01 (SUP1) or 25 mg/kg/day of BG02 (SUP2)	Immunomodulation: - ↑ Phagocytic activity of blood monocytes and neutrophils. - ↑ IL-2 and anti-ovalbumin antibodies production (antibody response). - ↓ Blood glucose in dogs with hyperglycemia.	(100)
Yeast-derived β-glucan (Biolex-Beta HP. Inter Yeast Poland).	0 (CON) vs. 7 mg/kg BW (SUP) daily for 6 weeks *Dogs with IBD	Immunomodulation: - ↓ IL6 and ↑ IL-10. Management of gastrointestinal disorders: - Most efficient treatment efficacy by producing the quickest therapeutic effect. - No recurrence of IBD at least for 6 months after the experiment.	(101)
Yeast-derived β-glucan (MacroGuard, Biorigin)	0 (CON) vs. 800 ppm of β-glucan (SUP) daily for 8 weeks. *Dogs with atopic dermatitis signs	Immunomodulation: - Numerical improvement in the scores of itching, redness, scaling, thickening, and stripping of skin, but notstatistical significance compared with CON. - Global improvement (sum of all the parameters) of the atopy index was 63% comparing SUP with CON. - ↑ Coat quality.	(102)
Yeast-derived β-glucan (MacroGuard, Biorigin)	0 (CON) vs. 800 ppm of β-glucan (SUP) daily for 8 weeks. *Dogs with osteoarthritis signs	Immunomodulation: - Numerical improvement in the scores of activity, stiffness, swelling of joing, lameness, and pain, but the differences between CON and SUP did not reach statistical significance.	(103)
Yeast-derived β-glucan (MacroGuard, Biorigin)	0 (CON) vs. 225 mg of β-glucan (SUP) daily for 4 weeks. *Experiment 1. No vaccine.* *Experiment 2. Vaccine challenge*	Immunomodulation: *Experiment 1:* - ↓ IgA and ↑ IgM serum concentrations. - ↑ - ↓ Saliva and tears IgA. Increase of saliva IgA after the end of the supplementation was also higher for SUP group. *Experiment 2:* - After vaccination, antigen-specific IgM increased similarly to total IgM. - Antigen-specific IgA remained nearly unchanged, whereas it was increased in CON.	(104)
Dried yeast	Different diets with different primary protein source: poultry by-product meal (CON), dried yeast product (SUP1, 30% of the diet), garbanzo beans (SUP2), green lentils (SUP3) and peanut flour (SUP4), for 14-day periods	Fecal microbiota: - ↑ β-diversity in SUP1. - ↑ Several genera belonging to Firmicutes. Hindgut fermentation products (fecal): - ↑ Acetate, butyrate and total SCFA. - ↑ Valerate. - ↓ Phenols and total phenol/indoles. Digestion: - ↓ DM, OM and AHEE ATTD, although still >80%. ↓ Metabolizable energy content. - ↑ Fecal output.	(105)
Dried yeast	0 (CON) or 0.19 g/kg BW (SUP) daily for 28–63 days	Obesity: - Removal of the yeast in hyperthyroid dogs resulted in lower intake and anorexia. *This study is about hyperthyroidism, B1 deficiency and yeast supplementation.	(106)

AHEE, acid-hydrolized ether extract; ALT, alanine transaminase; AST, aspartate transferase; ATTD, apparent total tract digestibility; BCFA, branched-chain fatty acids; BW, body weight; CF, crude fiber; CFU, colony forming units; CP, crude protein; CON, control group; DC, distal colon; DM, dry matter; GE, gross energy; IBD, intestinal bowel disease; IFN, interferon; Ig, immunoglobulin; IL, interleukin; LY, live yeast; MHC, major histocompatibility complex; MOS, mannan oligosaccharides; NFE, nitrogen-free extract; NDF, neutral detergent fiber; ME, metabolizable energy; OM, organic matter; PC, proximal colon; Ref., reference; SC, *Saccharomyces cerevisiae*; SCFA, short-chain fatty acids; SCFP, *Saccharomyces cerevisiae* fermentation product; SUP, supplemented group; TLR, toll like receptor; TNF, tumor necrosis factor; YCW, yeast cell wall; WBC, white blood cells.

**Table 3:** Main outputs of different studies on the use of live yeast *Saccharomyces cerevisiae* and by-products in cats.

Product	Dose	Results	Ref.
LY SC (strain MUCL 39885; Biosprint®, Prosol S.p.A.)	0 (CON) or 7.5 × 10^10^ CFU SC/kg feed daily for 35 days (SUP)	Digestion: - ↑ Fecal DM (day 28).	(73)
SCFP (XPC™, Diamond V)	0% (CON), 0.3% (SUP1) or 0.6% (SUP2) SCFP of diet for 25-day periods	Fecal microbiota: - ↓ *Clostridium perfringens*. Hindgut fermentation products (fecal): - ↑ Lactic acid. - ↓ Isovaleric in SUP1. Digestion: - ↑ ATTD of CF and ash. - ↓ GE in SUP2.	(107)
Yeast extract (Nupro®, Alltech Brasil Ltda.)	Exp. 1: 0% (CON) vs. 30% replacement by yeast extract (SUP). 10-day periods. Exp. 2: 0% (CON) vs. 2% (SUP1), 4% (SUP2), 6% (SUP3), 8% (SUP4) and 10% (SUP5) replacement. 15-day periods. Exp. 3: 0% vs. 2% replacement with yeast extract. 4 days.	Exp. 1: yeast extract as an ingredient: - According to ATTD, yeast extract is comparable to other protein sources. Exp. 2: yeast extract as a functional additive: - No changes. Exp. 3: yeast extract as a palatability agent: - Preference for control diet was observed.	(108)
Yeast extract (Nupro®, Alltech Brasil Ltda.)	0% (CON), 0.6% phosphoric acid (SUP1), 1.5% yeast extract (SUP2), combination of A + Y (SUP3) for 15-day period.	Digestion: - ↑ ATTD of DM and ashes in SUP3. - ↓ DM intake in SUP1 and SUP2.	(109)
Yeast extract	0 (CON), 0.2 (SUP1), 0.4 (SUP2) or 0.6% (SUP3) yeast extract in wet food	- Changes on the intestinal mucosa in SUP3 were noticed, related with an inflammatory response.	(110)
YCW	Exp. 1: 0 (CON), 0.2 (SUP1), 0.4 (SUP2) or 0.6% (SUP3) YCW in wet food, for 30 days. Exp. 2 (25 days): 0 (CON), 0.2 (SUP1), 0.4 (SUP2) or 0.6% (SUP3) YCW in dry food, for 25 days. Exp. 3: 0 and 0.4% YCW in wet food for 10 days.	Exp. 1: no changes. Exp. 2: ↑ ATTD of DM, linearly with dosage. Exp. 3: ↓ preference.	(111)
Yeast-derived MOS (Bio-MOS, Alltech Inc.)	0% (CON), 0.2% (SUP1) 0.4% (SUP2) and 0.6% (SUP) inclusion of YCW for 34-day periods	Fecal microbiota: - ↓ *Clostridium perfringens* (linear) and *Escherichia coli* (quadratic). - ↑ *Bifidobacterium* and *Lactobacillus* with dosage. Hindgut fermentation products (fecal): - ↑ Butyrate, valerate, total biogenic amines, putrescine, cadaverine and histamine increased with dosage. Digestion: - ↑ Ash ATTD of ash. Increased with YCW content.	(112)

AHEE, acid-hydrolyzed ether extract; ATTD, apparent total tract digestibility; BW, body weight; CF, crude fiber; CFU, colony forming units; CON, control group; CP, crude protein; DM, dry matter; GE, gross energy; LY, live yeast; OM, organic matter; Ref., reference; SC, *Saccharomyces cerevisiae*; SCFA, short-chain fatty acids; SUP, supplemented group; YCW, yeast cell wall.

### 4.1. Modulation of gut microbiota and hindgut fermentation

The addition of yeast by-products in dogs’ diet produces changes in gut’s bacterial population. Supplementation with SCFP has resulted in increases in the relative abundance of the phyla Firmicutes and/or Actinobacteria, and decreases of Fusobacteria (79, 81). Accordingly, use of dried yeast as primary protein source in a dog diet resulted in higher relative abundances of several genera belonging to Firmicutes phylum (105). An *in vivo* study simulating the canine intestinal microbial ecosystem also reported a decrease in Fusobacteria and an increase in Bacteroidetes with the addition of YCW (85).

At the genus level, *Bifidobacterium* increased with live yeast, SCFP, yeast hydrolysate, or MOS addition (5, 81, 82, 88, 94). *Prevotella* relative abundance increased while *Fusobacterium* decreased with higher doses of SCFP supplementation (81). Santos et al. (79) also observed reduced *Fusobacterium* and *Allobaculum*, as well as increased *Clostridium* with the inclusion of SCFP; in this same study, a level of 0.6% SCFP of the diet resulted in a decrease of *Prevotella* and an increase of genus *Collinsella*. Increases in genera considered beneficial and related with the production of SCFA, such as *Bacteroides*, *Faecalibacterium*, *Coprococcus*, and *Phascolarctobacterium*, were reported with the use of yeast products (75). Fecal *Lactobacillus* tended to be higher and total aerobes tended to be lower in dogs supplemented with MOS whereas supplementation with both FOS and MOS resulted in a tendency of lower total anaerobe concentration compared with control (95), although no impact on *Bifidobacterium* was noted in this case.

Increases in *Lactobacillus* and *Bifidobacterium* would likely yield increased lactic acid and short-chain fatty acids (SCFA), which could be the reason behind the lower fecal pH observed by some authors after the inclusion of yeast derivatives in dogs’ diets along with decreased fecal ammonia (27, 84). Bastos et al. (5) also observed lower fecal ammonia levels after an abrupt dietary change in dogs supplemented with live yeast compared to control ones. However, these finding have not been consistent as other authors have reported higher fecal pH (82, 90, 95) or no change (24, 74, 81, 86).

Short chain fatty acids are rapidly absorbed by colonocytes, thus determining their levels *in vivo* can be challenging, perhaps contributing to the varying results reported. Unaltered fecal concentrations of SCFA have been reported when yeast or yeast by-products were used (24, 74, 81, 95, 97). However, decreases in isovalerate and/or increases in propionate, butyrate, acetate and total SCFA when using yeast products have been reported by different authors (5, 75, 81, 95). Van den Abbeele et al. (85) also observed increases in acetate, butyrate and propionate when adding YCW in their *in vitro* experiment simulating the canine gut. Moreover, concentration of fecal phenol and/or total phenol + indole were lower in dogs supplemented with yeast products (5, 75, 81, 95). Total fecal biogenic amines decreased significantly in dogs receiving live yeast (5) (5). These results may indicate the possible beneficial effects of yeast and yeast by-products on the canine intestinal functionality.

Yeast cell wall supplementation in cats also led to increased fecal *Bifidobacterium* and *Lactobacillus*, and reductions of *Clostridium perfringens* (*C. perfringens*), *E. coli*, *Hafnia alvei*, and *Shigella* spp. (112). The inclusion of SCFP in cats’ diet reduced *C. perfringens* abundance, however no change for *Bifidobacterium*, *Lactobacillus*, or *E. coli* was observed (107). Supplementation with a microencapsulation of probiotic strains which include *S. cerevisiae* for 20 days resulted in fecal *Lactobacillus* count increase, but the effect of yeast cannot be isolated in this study (113). Aquino et al. (110) did not find differences in lactic acid bacteria counts nor clostridium-reductor when substituting up to 0.6% of the wet diet with yeast extract in cats.

There were no differences in cats’ fecal pH when SCFP or yeast extract was supplemented (107, 109). Matheus et al. (107) compared a control diet without SCFP with two different inclusion levels (0.3 and 0.6% of the diet), and failed to find differences in fecal pH, ammonia, or SCFA between treatments (except for isovaleric acid, which was lower in 0.3% SCFP inclusion group), However they did report higher fecal lactic acid concentration in cats supplemented with SCFP compared with control ones. On the other hand, lactic acid was not changed and neither propionate, isovalerate, acetate, isobutyrate, total SCFA, and total BCFA when YCW was included in the feed at 0.2, 0.4, or 0.6% of the diet (112). These last authors also found that the fecal concentrations of cadaverine, histamine, putrescine, and total biogenic amines increased linearly with the inclusion of YCW. Collectively, these studies present a lack of consistent responses to yeast or yeast derivatives, which may suggest strain specific impact, but that too will require additional assessments.

### 4.2. Immunomodulatory properties

Immunity is certainly an area of interest for functional ingredients and may be positively influenced by yeast intake, at least in dogs. Supplementation of dogs with YCW, alone or in combination with FOS, resulted in increased ileal and fecal IgA (86, 90, 95), indicating enhanced mucosal immunity. Some of these authors also found serum IgA increases of around 20% compared with control when including YCW at 0.45–0.5% of the diet. Lin et al. (81), on the other hand, did not find differences in serum IgA, IgE, IgG, and IgM when supplementing SCFP compared to control, but higher SCFP dosages led to higher IgE concentrations. However, these latter authors found that dogs supplemented with SCFP had increased population of antigen-presenting cells, including B-cells and monocytes expressing surface major histocompatibility complex class II. One hypothesis would be that fewer immune cells are needed when immune capacity is enhanced. Moreover, in the same study cells obtained from dogs supplemented with SCFP had a lower TNF-α secretion when stimulated with toll-like receptor (TLR) agonists compared to control dogs, suggesting an anti-inflammatory effect of SCFP. Accordingly, lower pro-inflammatory interleukin 6 (IL-6) and TNF-α, as well as higher blood monocyte phagocytic index, were shown by dogs receiving YCW in the study by Theodoro et al. (87). This increased phagocytic index was also observed with the inclusion of SCFP (79), althought others authors did not report any changes with yeast hydrolysate supplementation (82). The ingestion of yeast-derived β-glucan also produced lower levels of TNF-α in obese dogs (98), as well as lower serum concentrations of IL-6, increased anti-inflammatory IL-10, reduced haptoglobin (marker of inflammatory status), and tended to reduce gut permeability (97, 101).

Pawar et al. (92) found an enhanced cell-mediated immune response, CD4+ lymphocyte population and CD4+:CD8+ ratio in dogs supplemented with MOS. In contrast, Kroll et al. (91), did not observe changes in those cell communities when supplementing adult or senior dogs a second-generation MOS, rich in mannoproteins.

Studies with dogs involving stress challenges suggest some benefits related to the modulation of the immune system as well. Dogs supplemented with SCFP and challenged with exercise and transport stress had increased serum TNF-α and decreased IgE and IgG, although the vitality of these animals was higher (80). In another study involving transport stress challenge, SCFP addition caused stable or decreased cyclooxygenase-2 and myeloperoxidase mRNA expression, two key inflammatory mediators. These findings suggest a positive impact through suppression of innate immune cell activation against stress.

The supplementation of β-glucans derived from *S. cerevisiae* also influenced the humoral immune response to vaccination, affecting IgA and IgM responses (104), whereas Traughber et al. (99) did not observe any effect.

The modulatory effect of yeasts in the immune system is especially relevant in the treatment of some inflammatory diseases; in fact, β-glucan was superior to levamisole in the treatment of dogs affected by Inflammatory Bowel Disease (IBD) (101) and had promising results treating dogs with signs of canine atopy and osteoarthritis (102, 103).

Hematological changes due to yeast supplementation, similar to other parameters, vary greatly among the studies. Some studies have found an increase within the reference ranges in mean corpuscular volume and mean corpuscular hemoglobin concentration and no effect on white blood cells (WBC) counts in dogs fed live yeast (74). Others noted a decrease in WBC, monocyte and eosinophils counts in dogs given SCFP or YCW components (81, 90). Although these last authors did not find differences in lymphocyte counts, others found an increase (95) and a decrease (78, 94) when supplementing yeast derivatives to dogs.

Although the number of studies evaluating immune modulation in dogs is limited, even less is known regarding to cat. Matheus et al. (107) did not report any difference in cats supplemented with SCFP when comparing CD4+ or CD8+ lymphocytes, CD4+/CD8+ ratio, or phagocytosis index, among other parameters involved in immune response which also did not change. Hematological changes were neither observed when yeast extract was supplemented up to 10% in the diet for cats (108).

### 4.3. Inhibition of pathogens and management of gastrointestinal disorders

As previously discussed, supplementation of yeast and its derivatives can modulate gut microbiota, providing possible benefits. This subsection will go through the changes in potentially pathogenic microorganisms that also inhabit the gastrointestinal tract, and the role that yeast can have in the management of some gastrointestinal disorders.

Although *E. coli* can sometimes have a positive effect in the gut, such as stimulation of the immune system or vitamin K production, its proliferation is more often associated with a negative impact. In this regard, the supplementation of live yeast, autolyzed YCW and MOS from *S. cerevisiae* has resulted in lower counts of *E. coli* (5, 74, 90, 94), although some other authors have not found differences with addition of MOS, FOS, or the combination of both (95). Even in an *in vitro* experiment simulating the canine gut, adding YCW decreased the presence of the Enterobacteriaceae family, which includes *E. coli* (85). Another potentially harmful bacteria that could be modulated by yeast is *C. perfringens*. Whereas some authors did not find differences with MOS supplementation (95), other studies have reported higher *C. perfringens* counts with yeast extract supplementation but neither was accompanied by diarrhea (86, 90). However, Oba et al. (76) observed that *C. perfringens* abundance increased in control dogs and was stable for dogs fed SCFP for 21 days. Similarly, decreases in other species of *Clostridium* such as *Clostridium celerecrescens* and *Clostridium saccharolyticum* were observed in dogs fed YCW (88, 89). Bastos et al. (5) reported that control dogs, compared with dogs receiving a live yeast supplementation, showed upregulation in genes related to virulence factors, antibiotic resistance, and osmotic stress.

The use of *S. boulardii* in dogs with gastrointestinal disorders has also been studied, with promising results. One publication focused on the use of this yeast as a probiotic in dogs with diarrhea induced by lincomycin (114). Twenty-four dogs were given lincomycin intramuscularly (150 mg/kg BW/d) and three groups were formed (*n* = 8): (I) no *S. boulardii*, (II) *S. boulardii* (1,000 mg/d orally for 10 days) after lincomycin induced diarrhea onset, and (III) *S. boulardii* (1,000 mg/d orally for 10 days) together with lincomycin. The results showed that groups I and II had diarrhea and lower fecal SCFA concentrations, but not group III, and the duration of this gastrointestinal alteration was shorter in group II compared to control. Another publication used *S. boulardii* in dogs with chronic enteropathy (CE) (115). Four healthy dogs and 20 dogs with CE were used and *S. boulardii* was administered at a dose of 1 × 10^9^ CFU/kg BW orally in capsules every 12 h. In healthy dogs, the administration of *S. boulardii* for 10 days reached a steady state in 5 days and was eliminated completely 4 days after administration, without side effects. Dogs with CE received placebo or *S. boulardii* for 60 days: the yeast significantly improved clinical activity index, stool frequency, and consistency and body condition score over control dogs.

Lastly, yeast cell wall could also have a positive impact in recovering gut health. Dogs fed MOS and experimentally infected with different strains of enteropathogenic *E. coli* recovered faster than control ones, indicating an improvement in the defense system of the animals (93). On the other hand, yeast-derived β-glucan were superior against levamisole in the treatment of dogs affected by IBD, achieving a fast recovery with no recurrence, at least in 6 months (101).

### 4.4. Management of obesity

Components of yeasts such as β-glucan could play a role in obesity prevention and treatment, as obese dogs fed yeast-derived β-glucan had lower plasma basal glycemic values, serum cholesterol and triglyceride levels (98). Reduced serum total- and LDL-cholesterol concentration was also reported in dogs supplemented with MOS (92) and β-glucans (97, 98) from SC.

However, the potential effect of yeast on weight is not clear. Several studies reported no effect on feed intake or body weight (BW) in dogs (24, 73, 81, 86, 90, 97, 105). However, Stercova et al. (74) observed that dogs fed capsulated live yeast SC showed a higher increase of BW compared with the control ones, but the difference was very limited. Increases in intake ratio for diets containing yeast products when compared to diets without yeast have also been reported, although they were not accompanied by differences in dry matter intake or body weight increase (83, 84).

Lastly, a study involving dogs with hyperthyroidism showed that the cessation of yeast as a vitamin B1 source in hypothyroid dogs resulted in loss of appetite and anorexia (106).

### 4.5. Effects on digestion

Regarding the potential effect of yeast on digestive function and digestibility of nutrients, results in the literature are variable and sometimes contradictory, although the most common findings are decreases in CP ATTD and occasionally increases in fiber ATTD.

Live yeast supplementation had no significant effect on DM, ash, crude fiber (CF), CP and fat ATTD (5); only non-digestible fiber ATTD was higher in supplemented dogs (74). Santos et al. (79) also found increased ATTD of crude fiber when SCFP was added, but decreased ATTD of CP, nitrogen-free extract and metabolizable energy.

On the other hand, Middelbos et al. (90) tried different doses of YCW inclusion on the diet (from 0 to 0.65% of the diet, mostly MOS) and reported increases in DM, organic matter (OM), CP, and gross energy (GE) ileal digestibility compared to control animals but decreases in DM and CP ATTD. Similarly, YCW included as 1.4% of the diet resulted in reduced ATTD of DM, OM, CP, fat, and GE (89) and MOS as 5% of the diet reduced ATTD of DM, CP and nitrogen-free extracts (27). Addition of yeast extract or dried yeast also lowered ATTD of different macronutrients (84, 105). Protein excretion may result from increased bacterial growth and excretion due to increased fermentation, underestimating CP digestibility and, in turn, lowering OM and DM digestibility. However, other studies with various kinds of yeast products (MOS, β-glucans, SCFP and SC from Brewer’s yeast) did not find significant differences neither in ileal nor in apparent total tract nutrient digestibilities when compared to control diet (24, 75, 81, 83, 92, 94–96).

Fecal score was higher (wetter feces) in some studies supplementing SCFP, YCW or MOS to dogs when compared to control groups, although the values remained within the ideal score range (81, 89). Martins et al. (83) observed that dietary yeast inclusion of 30% of the diet resulted in diarrhea and soft feces but an inclusion of up to 15% was suitable, without changes in fecal consistency. However, the inclusion of a live SC yeast as in 7 × 10^10^ CFU/kg feed resulted in significant higher fecal dry matter, although the magnitude of the changes was of questionable biological relevance (73). Nevertheless, many of the other studies mentioned in this section did not find any differences in fecal score with the dietary inclusion of yeast products generally up to 5% of the diet, with the exception of the 12% inclusion of yeast extract in Kaelle et al. (84).

As for cats, regarding digestibility, there were no differences in nutrient and energy utilization when yeast extract was included in the feed up to 10% in the diet (108) or when yeast extract was added without an acidifying agent (109). Matheus et al. (107) also found relatively similar results, except for CF and ash digestibility, which were higher with SCFP inclusion, although the authors recommend taking these results with cautions as they warn that there may be calculation errors due to low fiber concentration of the diets. Yeast cell wall inclusion also resulted in improved DM and ash digestibility in cats (112).

### 4.6. Kidney and liver health

Dogs kidney and liver function assessed through serum urea, creatinine, alkaline phosphatase (ALP), and alanine transaminase (ALT) seems to be unaltered in some studies where dogs were supplemented with live yeast or yeast by-products (74, 81). However, the activity of ALT and aspartate aminotransferase (ASP) was increased in dogs supplemented with yeast hydrolysate during and after 28 days of supplementation (82). This same study showed increased serum urea levels in dogs receiving the hydrolysate in the post-treatment period whereas total protein, albumin, glucose, and ALP did not change significantly.

### 4.7. Potential antioxidant properties

Very few studies have assessed the effect of *S. cerevisiae* on the oxidative status of dogs, although some positive results have been obtained. Supplementation with SCFP at a dose of 500 mg/day decreased thioboarbituric acid reactive substance and increased total antioxidant capacity in serum of dogs exposed to exercise and transport stress, which could indicate an enhanced protection against oxidative damage (80). Wilson et al. (78) also measured markers and expression of genes related to oxidative stress in dogs fed SCFP compared to control ones and observed that superoxide dismutase enzyme increased from baseline and mRNA expression of catalase was lower. However, more investigation is necessary as another study of the same authors showed that SCFP-fed dogs increased malondialdehyde and 8-isoprostane levels due to transport stress (77). Lastly, the supplementation of MOS or SCFP (125, 250, and 500 mg/day) did not exert any effect on erythrocytic antioxidant indices when compared to the non-supplemented group (81, 92).

### 4.8. Skin and hair

To the knowledge of the authors, only two studies have investigated the effects of *S. cerevisiae* supplementation on skin and hair health of dogs. A tendency for higher change from baseline in sebum concentrations was observed in dogs fed SCFP that did not result in greasy hair coat (78). In this study, higher Transepidermal Water Loss (TEWL) in the ear region was also measured in supplemented dogs, possibly indicating enhanced skin integrity. However, TEWL in the back region was higher in control dogs but the authors of the study warn that this may be due to alterations during sampling. In fact, Beloshapka et al. (88) found no difference in the skin condition score in the tail region or any other skin and coat scores when dogs were supplemented with YCW.

### 4.9. Impact on palatability

Palatability tests comparing standard diets with diets including yeast products have resulted in a preference for the latter, probably due to the presence of glutamic acid and nucleotides, which can intensify flavors of the food (75, 81, 84). These results indicate that yeast or yeast by-products are well accepted by dogs, so their inclusion in commercial diets would not cause refusal and thereby negatively affect the food intake and body condition.

Regarding *S. boulardii*, there is one publication about the production of meat pet snacks containing this yeast that studied the production process, viability and acceptability of the snacks, showing that the addition of yeast had a good acceptance (116).

Contrary to dogs, in which the use of live yeast or derivatives seemed to increase palatability, results in cats are not uniform and tend to show worse palatability with yeast supplementation. No difference in feed intake compared to control group was found when evaluating the use of live yeast, SCFP or YCW (73, 107, 112).

On the other hand, Lima et al. (108) evaluated the use of yeast extract as a palatability agent observing that cats preferred the control diet over 2% inclusion yeast diet. Similar to Lima, Ogoshi et al. (109) found that the inclusion of yeast extract at 1.5% of the diet reduced feed consumption. Aquino et al. (111) reported lower palatability with wet diets that contained a 0.4% of YCW. Lower preference for yeast extract seems to be countered with the addition of sodium pyrophosphate; this blend was more preferred than control (117). Excess of umami taste and monosodium glutamate could reduce palatability and cats could have a lower threshold of acceptance. Moreover, processing may affect the flavor profile so thermal processes can decrease the flavor-enhancing effect and increase the acceptance of the ingredient. It is also hypothesized that the rejection of yeast extract is due to its high content in leucine as cats reject amino acids “bitter” for humans such as this one.

## 5. Concluding remarks and future perspectives

Pet humanization has led to an increase in the demand for functional ingredients in the pet food industry. Owners are more concerned about pet health and wellness and are willing to invest in diets with superior properties even if it means that they are more expensive, as long as their dogs and cats are healthier. The wide range of yeast-based products with potential to be used as prebiotic, probiotic, or postbiotic is of great interest at present, as they are becoming more related to the successful treatment of diseases. Based on the fact that the main scientific knowledge is gathered from human and livestock studies, further investigations are still required in pet species to better understand the mode of action, doses, safety, and guidelines for use of these products.

While the gut will probably remain the heartland of research, new clinical applications will continue to expand in other research areas including immune system, skin, nervous system, oral cavity, kidney, respiratory system, and weight management field. Thanks to significant and accelerating advances in biotechnologies and bioinformatics methodologies, new research will better characterize mechanistic insights into the mode of action of yeast and their derivatives, leading to a more targeted-oriented development of *S. cerevisiae*-based products.

## Author contributions

MM: Methodology, Writing – review & editing. LC: Methodology, Writing – review & editing, Supervision. SM-O: Methodology, Supervision, Writing – review & editing. AM: Writing – review & editing. OC: Writing – review & editing. AF: Writing – review & editing. AA: Writing – review & editing, Funding acquisition, Methodology, Supervision.
